# Introduction to Amniotic Membranes in Maxillofacial Surgery—A Scoping Review

**DOI:** 10.3390/medicina60040663

**Published:** 2024-04-19

**Authors:** Grzegorz Dawiec, Wojciech Niemczyk, Rafał Wiench, Stanisław Niemczyk, Dariusz Skaba

**Affiliations:** 1 Department of Paediatric Otolaryngology, Head and Neck Surgery, Department of Paediatric Surgery, Faculty of Medical Sciences, ul. Medyków 16, 40-752 Katowice, Poland; 2 Outpatient Clinic for Dental Surgery in Zabrze, University Dental Centre, Silesian Medical University Ltd. in Katowice, Pl. Akademicki 17, 41-902 Bytom, Poland; 3 Private Dental Practice NZOZ Stomatologia-Dawiec s.c., Ul. Witczaka 49/15, 41-902 Bytom, Poland; 4 Department of Periodontal Diseases and Oral Mucosa Diseases, Faculty of Medical Sciences in Zabrze, Medical University of Silesia, Pl. Traugutta 2, 41-800 Zabrze, Poland; rwiench@sum.edu.pl (R.W.); dskaba@sum.edu.pl (D.S.); 5 Municipal Hospital No. 4 in Gliwice, Zygmunta Starego 20, 44-100 Gliwice, Poland; stanislaw.niemczyk@szpital.gliwice.pl

**Keywords:** oral surgery, amniotic membrane, cleft palate, mucous membrane, vestibuloplasty, oroantral fistula, amnion, membranes

## Abstract

*Background:* Amniotic membrane (AM) holds significant promise in various medical fields due to its unique properties and minimal ethical concerns. This study aims to explore the diverse applications of the human amniotic membrane (HAM) in maxillofacial surgery. *Methodology:* A comprehensive search was conducted on databases, namely Google Scholar, PubMed, and Scopus, from January 1985 to March 2024. Articles in English, Polish, and Spanish were included, focusing on keywords related to amniotic membrane and oral surgery. *Results:* Various preservation methods for HAM were identified, namely fresh, decellularized, cryopreserved, lyophilized, and air-dried formats. Clinical studies demonstrated the efficacy of HAM in repairing oral mucosal defects, vestibuloplasty, oronasal fistula closure, cleft palate treatment, bone defect repair, and medication-related osteonecrosis of the jaw (MRONJ). Surgeon evaluations highlighted the ease of handling but noted challenges in suturing and stability during application. *Conclusions:* Amniotic membranes offer a versatile and effective option in maxillofacial surgery, promoting wound healing, reducing inflammation, and providing a scaffold for tissue regeneration. Further research, including randomized trials and comparative studies, is warranted to validate the efficacy and optimize the utilization of HAM in clinical practice.

## 1. Introduction

Amniotic membrane (AM) is a colorless and transparent membrane free of blood vessels, nerves, and lymphatics obtained from the innermost layer of the placenta [1,2]. It is one of the thinnest membranes (approximately 0.02–0.5 mm) in the human body [3]. The human amniotic membrane (HAM) consists of five distinct layers, namely the epithelium, basement membrane, compact layer, fibroblast layer, and spongy layer. The epithelium layer is positioned toward the amniotic fluid, while the outermost layer, the spongy layer, is connected to the vascular chorion [4]. The material can be harvested, sterilized, and preserved for use in various medical and dental fields [5]. Ethical concerns are minimal since it is obtained after informed consent following normal or cesarean deliveries [6]. However, the placenta derived from natural vaginal delivery may not be the optimal choice for AM isolation, due to potential structural changes and contamination with vaginal flora organisms [7]. The placenta must test negative for human immunodeficiency virus types I and II, human hepatitis virus types B (HBV) and C (HCV), syphilis, gonorrhea, toxoplasmosis, cytomegalovirus, and Treponema pallidum infections [7]. It is a cost-effective biomaterial that can be used as a scaffold for tissue engineering [6]. HAM contains numerous enzymes, growth factors, and cytokines, including prostaglandin synthase, epidermal growth factor, keratinocyte growth factor, hepatocyte growth factor, basic fibroblast growth factor, transforming growth factors, IL-6, and IL-8 [7]. HAM has several advantages as a tissue engineering material, including low immune repulsion and inflammatory response, a natural three-dimensional physical structure [8,9], the promotion of cell adhesion [10], antimicrobial activity, antiangiogenic properties, scarring inhibition, wound healing, epithelialization, and antitumorigenic properties [5,7]. The use of HAM grafts for the treatment of granulating wounds and burns has been in practice for over a century. Amniotic membrane was first used in ophthalmology in the 1940s. Following World War II, the use of amniotic membranes as a natural biological dressing for various types of wounds became widespread. Clinical experiences of numerous groups around the world have been published over the years [4]. Lawson et al. first described the use of HAM for treating oral mucosa defects in 1985 [11].

## 2. Materials and Methods

Google Scholar, PubMed, and Scopus databases were searched for articles published in English, Polish, and Spanish between January 1985 and March 2024. The following keywords were used: (“Amnion” OR “amniotic membrane” OR “amniotic mesenchymal stem cell” OR “amniotic epithelial cells”) AND (“oral mucosa” OR “oral surgery” OR “maxillary” OR “ jaw” OR “vestibule” OR “bone regeneration” OR “oral cavity” OR “tongue” OR “cleft palate”). Furthermore, additional articles were added after scanning manually the reference lists of all publications included. Articles describing animal studies and articles not describing amniotic membrane preservation methods were excluded.

## 3. Methods of HAM Preservation

Currently, the usual storage formats are cryopreserved, lyophilized, or air-dried [12].

### 3.1. Fresh Amniotic Membrane (FAM)

AMs can be used when it is fresh, but in most countries, legislation requires AMs to be stored for 6 months until a negative HIV screening result is confirmed [7]. FAM, with its native structure, preserved growth factors, and living stem cells, is a promising amniotic product for tissue engineering [13]. It has been reported that both fibroblast and epithelial cells remain alive after isolation fromFAMs, while these cells lose their viability after isolation from cryopreserved AMs (CAMs) [14]. The main disadvantages of fresh AMs are their short storage time and slight inflammatory reaction [7,13].

### 3.2. Decellularized Amniotic Membrane (DAM)

The process of decellularization can lead to a significant reduction in the thickness, mechanical properties, and immunogenicity of the amniotic membrane (AM) while increasing its degradation rate and safety [7,15]. A decellularized amniotic membrane (DAM) is a better choice for corneal and skin wound healing than a fresh amniotic membrane (FAM) as it can support cell growth and adhesion. The aim of this class of techniques is to remove cellular debris, resulting in a matrix with a high percentage of functional groups for cell attachment and migration [16].

Ethanol is a readily available decellularization agent. Although not a potent decellularization agent, it is very fast and safe. Successful decellularization can be achieved by further aggressive scraping after ethanol treatment. For instance, the decellularization of AM with 20% ethanol for 30 s, followed by aggressive scraping, revealed successful decellularization with maintained ECM composition, intact basement membrane, and growth factor expression [7].

### 3.3. Cryopreserved Amniotic Membrane (CAM)

Cryopreservation is the most common method for preserving AMs. There are several published clinical studies that confirm the safety, effectiveness, and advantages of this preservation method. Various studies have confirmed the potential of cryopreserved AMs in managing different skin wounds [7,17]. The cryopreservation of AMs in 10% DMSO (dimethyl sulfoxide) or 50% glycerol is a standard method for preserving AMs for tissue engineering and regenerative applications in the European Union. AMs can be cryopreserved by glycerol or DMSO at −80 °C for up to 12 months [7,18].

### 3.4. Lyophilized Amniotic Membrane (LAM)

Lyophilization, also known as freeze-drying, is a reliable method for preserving AMs for long-term storage, even at room temperature. The histological structure of lyophilized AMs (LAMs) remains unchanged. However, it has been shown that LAMs lose some of their total protein and growth factor concentration during lyophilization when compared to FAMs and CAMs. Despite this, LAMs have superior properties, including safety, higher graft take, longer shelf life, and ease of handling and transportation when compared to CAMs [7]. Dehydrated or lyophilized hAMs, sterilized by gammarays, can be stored at room temperature for several years. These preservation methods have been observed to result in minimal alterations in the biological properties of hAMs [19].

### 3.5. Air-Dried Amniotic Membrane (AD-AM)

AD-AM is manufactured following a standardized procedure [19]. The procedure includes a 4 h sterilization step at room temperature, using a mixture of peracetic acid (PAA) and ethanol. Afterward, the membrane undergoes three rinses with sterile physiological saline solution to eliminate any remaining PAA/ethanol residue [20].

Pieces of AM are kept at room temperature under a biohazard hood with the epithelial side facing downwards and the spongy layer facing upwards. They are then exposed to air for varying time periods ranging from overnight to 24 h [12,20]. The PAA/ethanol sterilization method used in AD-AM effectively deactivates bacteria, fungi, and viruses. Ethanol, in particular, denatures proteins and may contribute to changes in the basement membrane as well as the denaturation of soluble growth factors and cytokines. Furthermore, it has been reported that the process of air-drying during production may result in a decrease in protein content. Specifically, the lyophilized AM, which involves air-drying, has been found to contain less protein than Cryo-AM [20].

### 3.6. Sterilization of Amniotic Membrane 

Sterilization is a crucial step in minimizing the risk of infection transmission by the AM. Gamma irradiation and PAA are two commonly used sterilization agents for AMs. The combination of freeze-drying and gamma irradiation is an effective preservation and sterilization method that has been widely used for AMs. Research has shown that gamma irradiation in doses of 25–50 kGy does not affect the water absorption capacity, chemical and structural properties, or water vapor transmission rate of the AM. The study showed that gamma irradiation of glycerol preserves the AM at doses of 25 kGy or less without affecting its morphology or appearance [7].

## 4. The Use of Amniotic Membranes in Maxillofacial Surgery

### 4.1. Repair of Oral Mucosal Defects 

The oral mucosa is composed of two tissue layers: the superficial epithelium and the underlying lamina propria. Its main function is to act as a barrier against exogenous substances and pathogens. During development, the interactions between the stem/progenitor cells of the epithelium and mesenchyme are crucial for the morphogenesis of the oral mucosa [21]. The oral mucosa can be damaged by progressive cancerous lesions, during the excision of lesions and harvesting of tissue grafts, as shown in the articles in Table 1. The human amniotic membrane has been used to cover wounds after surgical removal of leukoplakia, pre-neoplastic and neoplastic lesions in six studies, after maxillectomy in one study, after the excision of submucous fibrosis in twostudies, and as a tissue graft harvesting site covering in two studies. 

Hazarika et al. are the only ones who have used the properties of amniotic membranes in post-maxillectomy treatment. They used its lyophilized form for this purpose. The follow-up time was 2 months during which the clinical picture was assessed subjectively. Despite the large defect in the amniotic membrane, the entire defect became completely epithelialized, so there was no need for an obturator for the patient [22].

Lai et al. (1995) used a fresh amniotic membrane to treat mucosal defects after the excision of submucous fibrosis. Another group, however, was treated using a buccal fat pad. After a 24-month follow-up period of 150 patients, they indicated that HAM improved interincisal distance compared with pharmaceutical therapy but decreased compared with skin or buccal fat pad grafts [23]. In contrast, a different conclusion was reached by Sharma et al. (2022), who, based on the results offive patients with 6 months of follow-up, indicated that in comparison to the buccal fat pad flap, HAM is better for oral reconstruction in terms of infection, graft failure, MMO, inflammation, and pain [24].

Mario et al. (2019), in their case report based on subjective clinical observations, described the coverage of a surgical wound after harvesting a tissue graft for gingival augmentation. They used a cryopreserved amniotic membrane for this purpose, and the follow-up time was 18 months. It was reported that there was wound closure as early as one week after the CAM covering procedure. There were no signs of infection, and composite re-epithelialization could be seen. In addition, there was no need for the patient to take pain medication [25]. The study by Kadkhoda et al. (2020), which appeared a year later, used the same type of amniotic membrane, for the same procedure, while the study group consisted of 27 patients, and the follow-up time was 21 days. During this time, a higher color match score was observed than the control group, where the amniotic membrane was not used. The pattern of pain relief was better in the test group compared with the control group, especially in the first days [26].

Tsuno et al. described two cases of using amniotic membranes to dress surgical wounds exposing bone created after the excision of precancerous and cancerous lesions. One week after surgery, the wound surface appeared smooth and glossy, while full epithelialization was noted after 6 weeks. In one of the cases, the VAS (0–10) relating to postoperative pain did not exceed 1, and in the other case, mild hyperalgesia was noted, while pain did not exceed level 3 on the VAS. Because of the hyper-dry AM’s transparency and good adherence to the irrigated wound surface, the wound tissues could be seen clearly through the membrane [27]. 

Two years earlier, Arai et al. performed wound protection after the surgical treatment of mucosal lesions with hyper-dry amniotic membranes in 10 patients. In five of them, the lesion was excised from the tongue; in four cases, treatment withthe AM was very useful, and in one case, it was useful. The remaining five patients had the lesion excised from the buccal region. In three of these patients, the AM was very useful, and in two, it was useful. In contrast, in none of these patients did the lesion expose bone. The hyper-dry AM showed good operability in all cases. It was not only easy to cut and shape but also adhered well to the irrigated wound surface. Hemostasis was generally effective, and no bleeding was observed in cases involving the buccal mucosa. Among the cases involving the tongue, only one showed bleeding after the removal of the pressure dressing. The bleeding was minor and could be stopped by applying pressure using an absorbable local hemostat [28]. 

Kar et al. chose to use cryopreserved AMs in their 34 patients for the repair of oral mucosal defects. Of the 34 lesions, 29 were leukoplakia, 3 were erythroplakia, and 2 were verrucous hyperplasia. The most common location of the lesions was the buccal (13) and alveolar mucosa (7). In six cases, the lesion involved both of these sites. Postoperative pain improved in 79% of cases on day 5 (13 patients had mild pain, and 14 patients were asymptomatic). Regarding sensory response, 28 patients (82%) reported normal sensation, while 6 (18%) still had altered sensation after six months. Among objective parameters, postoperative swelling improved in 88% of cases on day 5 (22 patients had mild swelling, and 8 patients were asymptomatic). Regarding oral opening after 6 months, 24 patients (71%) had good mouth opening, and 10 patients (29%) had fair mouth opening. None of the patients had poor oral opening [29].

Using a freeze-dried amniotic membrane, Hazarika et al. (2022) treated mucosal defects after the excision of precancerous lesions in 15 patients. After a two-month follow-up period, a change in mouth opening was recorded as none or little in 13 patients and serious in 2 patients. Mucosal suppleness was rated as fair in all the patients. No wound infection or allergic reaction was noticed in any of the patients. The feeding situation was rated as fair in all the patients. Epithelialization was rated as good in 13 cases and fair in 2 cases. Pain control was recorded as good in eight cases and fair in seven cases. In contrast, immediately after the procedure, hemostasis was rated as good in 5 cases and fair in 10 cases [30].

Sikder et al. published a case report in which they used an oven-dried human amniotic membrane to cover an oral mucosal defect from the buccal region. The follow-up time was six months, but the complete healing of the wound occurred after just four weeks. After the first week, a healthy granulation tissue was formed, and after the second week, epithelialization was completed [31].

A study on the clinical application of amniotic membrane as a biological dressing in the oral cavity and pharyngeal defects after tumor resection was published by Khademi et al. in 2013. The number of patients was 50. The anatomic location of tumors wasthe tongue in 34 patients, the floor of the mouth in 6 patients, 5 in the buccal area, and 2 in the retromolar area.

In all cases, the complete adherence of the amniotic membrane (AM) to the wound was observed. None of the patients reported any sensation of a foreign body, and they expressed comfort with the intraoral grafting of AM. There were no reports of systemic or local allergies in any of the cases. Pain relief was deemed satisfactory in all instances. Both granulation and epithelialization processes showed positive progress in all 50 patients. The membrane proved highly effective in 40 patients and effective in 10 patients. Furthermore, the membrane demonstrated great utility in all patients [32].

**Table 1:** Studies on the use of amniotic membranes in the repair of mucosal defects. F—female, M—male, AM—amniotic membrane, HAM—human amniotic membrane, VAS—visual analog scale.

Author Year	Patients	Sex	Age Range	Indications	Treatment	Evaluation Methodology	Results	Follow-Up Period
Tsunoet al., 2014 [27]	2	1F 1M	43–74	Mucosal defect after excision of precancerous and cancerous lesions	Hyper-dry AM	Subjective clinical observation	One week after surgery, the wound surface appeared smooth and glossy. Epithelialization of the entire wound was observed approximately six weeks after surgery, with no signs of rejection or excessive inflammatory reaction.	−18 months −3 years
Araiet al., 2012 [28]	10	6F 4M	54–89	Mucosal defect after excision of precancerous and cancerous lesions	Hyper-dry AM	Scoring index of hAM usefulness and its effectiveness (operability, hemostatic status, pain relief, feeding situation, epithelialization, scar contracture, and safety)	There were no adverse reactions reported. hAM was highly effective in three patients and effective in seven patients. It was also reported to be extremely useful in seven patients. The operability of hAM was good.	3–36 months (mean 20.9)
Kar et al., 2014 [29]	34	8F 26M	21–60	Mucosal defect after excision of oral precancerous lesions	Cryopreserved AM	Clinical measurement (swelling, epithelialization, oral opening, and mucosal suppleness)—Subjective parameters (pain and sensory response)	- After 3 months, all patients showed goodepithelialization. - After 6 months, oral opening was good in 24patients and fair in 10 patients. - Notably, 28 patients reported normal sensation and six stillhad altered sensation after 6 months.	6 months
Kadkhoda et al., 2020 [26]	27	13F 14M	18–70	Surgical wound on palatal site after harvesting graft for gingival augmentation	Cryopreserved AM	Postoperative pain on the VAS. Number of analgesics taken. Evaluation of photographs taken 7,14 and 21 days postoperatively	Higher color match scores than the control group. The pattern of pain relief was better in the test group compared with the control group, especially in the first days	21 days
Hazarikaet al., 2022 [30]	15	4F 11M	32–65	Mucosal defect after excision of oral precancerous lesions	Lyophilized AM	The effectiveness of the lyophilized AM was scoring the following parameters operability, hemostatic status, pain, feeding situation, epithelialization, change in mouth opening, mucosal suppleness, and safety.	Hemostasis was rated as good in 5 cases and fair in 10 cases. Pain control was recorded as good in 8 cases and fair in 7 cases. The feeding situation was rated as fair in all patients. Epithelialization was rated as good in 13 cases and fair in 2 cases. Changes in mouth opening were evaluated as none or little in 13 patients and serious in two patients. Mucosal suppleness was rated as fair in all patients. No wound infections or allergic reactions were noticed in any of the patients.	2 months
Mario et al., 2019 [25]	1	1F	38	Surgical wound on palatal site after harvesting graft for gingival augmentation	Cryopreserved AM	Subjective clinical observation	Closure of the wound one week after application of HAM—absence of infection—complete reepithelialization—no pain in few days following procedure—patient had not taken painkillers	18 months
Sikder et al., 2010 [31]	1	1F	50	Oral mucosal defect after surgical excision of leukoplakia	Dried AM	Subjective clinical observation	After the first week, healthy granulation tissue had formed. By the second week, epithelialization was complete, and by the fourth week, wound healing was observed.	6 months
Lai et al., 1995 [23]	150	5F 145M	17–68	Mucosal defect after excision of submucous fibrosis	(1–3)Pharmaceutical therapy (4) Skin graft (5) Fresh AM (6) Buccal fat pad graft	Measurement of interincisal distance	The study found that HAM resulted in an improved interincisal distance comparedto pharmaceutical therapy, but a decreased interincisal distance compared to skin or buccal fat pad grafts.	24 months
Sharma et al., 2022 [24]	5	No data	>18	Mucosal defect after excision of oral submucous fibrosis	(1) Lyophilized AM (2) Buccal fat pad	Pain assessment scale (VAS) Healing Index by Laundry et al. Subjective clinical observation	Compared to the buccal fat pad flap, the HAM is a better option for oral reconstruction in terms of infection, graft failure, maximum mouth opening, inflammation, and pain.	6 months
Hazarika et al., 2017 [22]	1	No data	No data	Maxillectomy defect coverage	Lyophilized AM	Subjective clinical observation	In the first week, a white necrotic slough formed, and in the second week, slight hyperemic mucosal tissue was observed. Within two months, the entire defect had completely epithelialized, eliminating the need for an obturator, and the patient’s nasal twang was no longer present.	2 months
Khademi et al., 2013 [32]	50	10F 40M	20–80	Oral cavity and pharyngeal defects after tumor resection	AM preserved in glycerol	Granulation tissue formation and epithelialization evaluation	In all cases, complete adherence of AM to the wound was observed without any reported allergic reactions, either systematic or local. The membrane was highly effective in 40 patients and effective in 10 cases.	2–20 months

### 4.2. Vestibuloplasty

In cases of alveolar ridge resorption in the edentulous mandible, there is a reduction in the surface area of the attached mucosa on the ridge. In such situations, the relationship between the mucosa and the muscles near the area where the complete denture is seated becomes crucial for prosthesis retention and stability. One approach to enhance prosthesis stability under these circumstances is to perform a procedure known as vestibuloplasty, which involves lowering the connection point between the mucosa and muscle, thereby deepening the vestibule [33]. The human amniotic membrane (hAM) serves as a remarkable biological graft, possessing distinct properties that include antiadhesive and bacteriostatic effects. It provides wound protection, minimizes pain, and promotes effective epithelialization. Furthermore, an essential attribute of hAM is its lack of immunogenicity. Its exceptional biological and biophysical properties, coupled with its wide availability and relatively low preparation, storage, and utilization costs, contribute to its superior performance compared to other grafts. Recent investigations have revealed that the hAM serves as an abundant source of stem cells capable of differentiating into various cell types, including chondroblasts, osteoblasts, adipocytes, myocytes, and neuronal cells [34].

The pioneering work of Güler et al. in 1997 introduced the application of the human amniotic membrane in mandibular vestibuloplasty. In their study, the hAM was grafted and sutured onto the exposed periosteum in a cohort of 20 patients. Comprehensive measurements of blood flow and meticulous clinical observations were conducted to evaluate the outcomes. The findings revealed that the hAM exhibited a significant angiogenic effect, demonstrated by a rapid increase in blood flow within the graft region. Remarkably, by the 14th day, the hAM had macroscopically disappeared, and complete epithelialization was observed three weeks postsurgery [35].

Both Kothari et al. (2012) and Sharma et al. (2011) conducted similar studies involving vestibuloplasty procedures in 10 patients using fresh amniotic membranes. Additionally, the follow-up time in both studies was 3 months, and the method for evaluating the results was vestibular depth measurement. According to their results, Sharma et al. reported a deepening of the vestibule by 4–6 mm, while Kothari et al. observed a reduction in the depth of the labial vestibule ranging from 17% to 50%. At the baseline, the average depth of the vestibule was 3.3 mm, and 3 months after surgery, it was 10 mm [34,36]. In 2004, Samandari et al. measured the vestibular depth in seven patients after using a fresh amniotic membrane in a vestibuloplasty procedure after a period of 6 months. There was a reduction in vestibular depth on the buccal side ranging from 17% to 40% [37]. Babaki et al. were the only researchers to undertake a comparison of vestibuloplasty efficacy between amniotic membranes (cryopreserved) and the acellular dermal matrix. They primarily examined vestibular depth and flow cytometry in their metological evaluation. The follow-up time of the patients was 3 months.From their results, we can see that there was no significant difference in the relapse of vestibular depth between the two grafts at different time intervals. However, the frequency of wound-infiltrating macrophages (CD68+ cells) was significantly higher in areas covered by the ADM after 3 and 7 days [38].

Tsunoet al. also described a single case report in which they used a hyper-dry AM for vestibuloplasty, where the evaluation method was subjective clinical observations over a 3-year follow-up study. Although the wound surface was varied, membrane adherence was good. After removing the splint one week after surgery, the wound surface appeared smooth and glossy. Favorable epithelialization of the wound was observed, with no rejection or excessive inflammatory reaction. Sufficient keratinized gingiva was present approximately six weeks after vestibuloplasty [27].

All of the authors concluded that human amniotic membranes are suitable materials for performing vestibuloplasty. Table 2 summarizes the information gathered from these studies.

### 4.3. Oronasal Fistula

There are only two studies on a total of 13 patients that involved damage to the sinus membrane. Rohelder et al. used five layers of a cryopreserved amniotic membrane for this purpose, while Holtzclaw used a dehydrated human amnion/chorion membrane. Control studies consisted only of subjective clinical examination. The complete closure of the oronasal fistula was observed in all patients. Table 3 summarizes the information gathered from these studies.

### 4.4. Cleft Palate

The issue of treating cleft palate with amniotic membranes was raised in 2015 by Tsuno et al. in a study on rats. Their results suggest that the hyper-dry amniotic membrane (HDAM) is a suitable new dressing material for use in the treatment of cleft palate [41]. In 2023, Fujiwara et al. published an article on the same issue, but this time, the study population included 16 patients with a mean age of one year and nine months. One-stage pushback palatoplasty was performed. The remaining raw wound after surgery was covered by an HDAM and a plastic cover plate, which was removed one week after surgery. Five days after the surgery and upon removal of the cover plate, all patients were able to eat adequately. None of the patients experienced a persistent fever or allergic reactions. Immediate ingestion was possible for all patients without any instances of postoperative bleeding. During the follow-up period, there were no occurrences of secondary hemorrhages. There were no instances of wound separation along the midline of the palate after the surgery. After the cover plate was removed, no infections were observed. Throughout the follow-up period, none of the patients experienced severe scar formation or wound contracture. There were no instances of hemorrhage, excessive epithelialization, or scar contracture in any of the patients. Table 4 provides a summary of the data collected from the study.

### 4.5. Bone Defect Repair

Many authors have obtained very good results using amniotic membranes to treat bone defects as barrier membranes. The bone thus obtained after augmentation was characterized by a lower level of resorption and better quality and additionally allowed for keratinized gingival growth. A detailed description of the studies considered is presented in Table 5.

### 4.6. Medication-Related Osteonecrosis of the Jaw (MRONJ)

Medication-induced osteonecrosis of the jaw (MRONJ), also referred to as medication-related osteonecrosis of the jaw, is a rare but potentially severe condition. Initially, it was primarily associated with the use of bisphosphonate (BP) medications. However, recent research has revealed that individuals receiving various types of medications, including the receptor activator of nuclear factor kappa-B ligand inhibitors (such as denosumab) and antiangiogenic agents, are also at risk of developing MRONJ. This expanded understanding highlights the broader range of medications that can contribute to the occurrence of MRONJ [46]. To date, there have been only five papers describing the effect of amniotic membranes on the treatment of MRONJ on a total base of 68 patients. MRONJ recurrence was detected in only 6 patients, while full recovery was noted in 57 of them, and most patients had considerable alleviation from pain and infectious signs shortly after surgery. All recurrences were discovered within 1 to 6 months of the follow-up. Cryopreserved amniotic membranes were used in all studies, and patients had all three stages of MRONJ. The follow-up time, depending on the study, was between 3 and 42 months, and all of the patients also underwent radiological follow-up. Table 6 provides a summary of the data collected from these studies.

### 4.7. Repair of Nasal Septal Perforation

Farhadi Shabestari et al. described 12 cases in which amniotic membranes were used to close nasal septal perforations (NSPs). After 3 months, the successful closure of the defect was observed in 10 out of 12 patients, resulting in an 83% success rate. The closure rate was 100% for defects smaller than 1 cm and 80% for defects sized 1–2 cm. All of the cases, including the two cases with reperforation, reported the elimination of all symptoms associated with NSPs during the postsurgical period [52]. A detailed description of the study is presented in Table 7.

## 5. Evaluation of Amniotic Membrane Use by Maxillofacial Surgeons

Surgeons who have worked on amniotic membranes have assessed that, in general, it is an easy material to operate on. The human amniotic membrane demonstrated remarkable strength during manipulation, particularly when detached from the nitrocellulose support, as indicated by surgeons. However, the fragility of the HAM posed challenges during suturing, with stitches occasionally causing cracks upon tightening and a propensity for self-folding, impeding the suturing process. Upon thawing and rinsing, the wet HAM displayed a tendency to fold inward, further complicating manipulation and separation from the nitrocellulose support. Surgeons unanimously agreed that the HAM was more manageable when it remained attached to its support and recommended this approach for the ease of cutting. Two out of five surgeons reported that folding the HAM facilitated its application to the surgical site, albeit resulting in increased thickness and loss of orientation. Conversely, other surgeons found that utilizing the HAM in a flat state offered two notable advantages: maintaining HAM orientation and effective placement between the bone and mucosa. During the suturing phase, three out of five surgeons noted the HAM’s relative instability at the surgical site, with a tendency to elevate between stitches when approximating the mucosal edges. However, it remained sufficiently stable to prevent expulsion from the surgical site. In contrast, two surgeons reported satisfactory stability during suturing, with no observed movement or oral exposure of the HAM. Overall, these findings provide valuable insights into the manipulation and handling characteristics of the HAM, highlighting the importance of proper technique and support attachment to optimize surgical outcomes [53].

## 6. Discussion

Amniotic membranes have found their way into the field of maxillofacial surgery, especially in recent years. The number of applications is steadily increasing, and research results are promising. The authors of this review have tried to focus on presenting a broad spectrum of applications of amniotic membranes and presenting information related to them, making it easier to introduce readers to this field. However, this review has its weaknesses, which should be highlighted. This is not a systematic review but a scoping one. Studies on small numbers of patients, including case studies, were also included in this review. The methodology of the reviewed studies in the same area was not always consistent, which translates into problems in accurately comparing results. Despite the shortcomings of this review, its strengths are also worth highlighting. The review was written based on both the first studies on HAM and the most current literature. In addition, studies on animals were excluded. Three authors were involved in the process of collecting and qualifying articles in order to eliminate the possibility of omitting relevant articles. The authors presented the results, treatment, study methodologies, outcomes, and follow-up period in detailed tables, given the scoping nature of the study. The authors suggest that future studies should focus on randomized trials comparing amniotic membranes to other materials or treatments to accurately assess the usefulness of amniotic membranes in specific procedures. Studies comparing the method of storing HAM should also be conducted to determine the most favorable solution.

## 7. Conclusions

Amniotic membranes have ideally found their application in maxillofacial surgery. Their anti-inflammatory and anti-immunological properties allow for a wide range of applications. None of these studies showed any adverse effects from the use of HAMs, and all showed satisfactory results. The authors repeatedly stressed that, due to its physical properties, HAM is a material with good operability. The HAM used in mucosal defects facilitates good hemostasis, epithelialization, high similarity in colormatching to surrounding tissues, and the preservation of sensation, and further reduces discomfort associated with healing. It allows for the effective closure of oronasal fistula and nasal septal perforation and can additionally be used in cleft palate procedures. It can also be used in the treatment of bone defects, augmentation, and maxillomandibular reconstruction. It allows for undisturbed healing and a larger bone volume of better quality, thus facilitating future implantation. It can also find its application in the treatment of any of the stages of MRONJ. Thanks to its properties, it allows for the exposed bone to be covered for a long period of time, reducing pain among patients and preventing infection of the necrotic site.

## Figures and Tables

**Table 2:** Studies on the use of amniotic membranes in vestibuloplasty. F—female, M—male, AM—amniotic membrane, CAM—cryopreserved amniotic membrane, ADM—acellular dermal matrix.

Author Year	Patients	Sex	Age Range	Indications	Treatment	Evaluation Methodology	Results	Follow Up Period
Babakiet al., 2021 [38]	28	15F 13M	Mean 58	Vestibuloplasty	(1) CAM (2) ADM	- Vestibular depth - Flow cytometry	There was no significant difference in the relapse of vestibule depth between the two grafts at different time intervals. However, the frequency of wound-infiltrating macrophages (CD68+ cells) was significantly higher in areas covered by ADM after 3 and 7 days.	3 months
Güler et al., 1997 [35]	20	9F 11M	42–84	Vestibuloplasty	CAM	Measurement of blood flow by the 133Xe clearance technique	- Angiogenic effect of hAM - Complete epithelialization after 21 days - No adverse reaction	1 month
Kothari et al., 2012 [34]	10	5F 5M	35–70	Vestibuloplasty	Fresh AM	Vestibular depth	After 3 months of follow-up, the depth of the labial vestibule decreased by 17% to 50%.	3 months
Samandariet al., 2004 [37]	7	4F 3M	Mean 63	Vestibuloplasty	Fresh AM	Vestibular depth	After 6 months of follow-up, the reduction in the depth of the buccal vestibule ranged from 17% to 40%.	6 months
Sharma et al., 2011 [36]	10	3F 7M	Mean 58.5	Vestibuloplasty	Fresh AM preserved in 85% glycerol	Vestibular depth	Gain of 4–6 mm after 3 months was noted.	3 months
Tsunoet al., 2014 [27]	1	1F	74	Vestibuloplasty	Hyper-dry AM	Subjective clinical observation	- Although the surface of the wound was varied, membrane adherence was good—One week after surgery, the wound surface appeared smooth and glossy upon removal of the splint. - Favorable epithelialization of the wound was observed, with no signs of rejection or excessive inflammatory reaction. - Approximately 6 weeks after vestibuloplasty, sufficient keratinized gingiva was present.	3 years

**Table 3:** Studies on the use of amniotic membranes in oronasal fistulas. F—female, M—male, AM—amniotic membrane, CAM—cryopreserved amniotic membrane, dHACM—dehydrated human amnion/chorion membrane.

Author Year	Patients	Sex	Age Range	Indications	Treatment	Evaluation Methodology	Results	Follow Up Period
Rohleder et al., 2013 [39]	4	2F 2M	21–51	Oronasal fistula	CAM	Subjective clinical observation	- Complete closure of the oral epithelium - No adverse reaction	76 days
Holtzclaw 2015 [40]	9	No data	No data	Perforation during sinus augmentation before implantation	dHACM	Subjective clinical observation	Repair was achieved with a single amnion-chorion barrier in each of the Schneiderian membrane perforations.	42 days

**Table 4:** The study on the use of amniotic membranes in cleft palates. F—female, M—male, HDAM—hyper-dry amniotic membrane.

Author Year	Patients	Sex	Age Range	Indications	Treatment	Evaluation Methodology	Results	Follow Up Period
Fujiwara et al., 2023 [42]	16	8F 8M	1–3	Cleft palate	HDAM	The cover plate was removed one week after surgery. The following parameters were monitored: temperature, feeding, allergic reactions, postoperative bleeding, re-epithelialization, wound dehiscence, and infection.	All patients were able to ingest food adequately 5 days after the operation. None of the patients experienced fever, allergic reactions, or infections. There was no postoperative bleeding observed during ingestion, nor were there any secondary hemorrhages during follow-up. Additionally, no postoperative wound dehiscence on the midline of the palate was observed, and there was no severe scar formation or contracture of the wound during the follow-up period.	31.2 months

**Table 5:** Studies on the use of amniotic membranes in the repair of bone defects. F—female, M—male, BFSC—buccal fat pad-derived stem cells, AM—amniotic membrane, HAM—human amniotic membrane, CBCT—cone beam computer tomography, VAS—visual analog scale, dPTFE—dense polytetrafluoroethylene membranes, FAM—fresh amniotic membrane, ACM—amnion chorion membrane.

Author Year	Patients	Sex	Age Range	Indications	Treatment	Evaluation Methodology	Results	Follow Up Period
Yu et al., 2022 [43]	3	2F 1 No data	45–48	Horizontal ridge augmentation	Not specified	Not specified	- Uneventful healing - Increased bone volume - Gain of keratinized tissue	4 months
Akhlaghi et al., 2019 [44]	9	6F 3M	Mean 25.87	Maxillomandibular reconstructions	(1) BFSC + lyophilized AM (2) Lyophilized AM	- Histological analysis - Clinical assessments - Radiological examination with CBCT	The combined use of horizontal alveolar distraction osteogenesis (HAM) with mesenchymal stem cells has the potential to enhance bone regeneration in the horizontal dimension. Additionally, the use of bone formation and stabilization devices (BFSCs) can reduce the amount of harvested autogenous bone and minimize secondary bone resorption.	5 months
Hassan et al., 2017 [45]	9	3F 6M	34–71	Alveolar ridge preservation	(1) FAM (2) dPTFE	- Histological analysis - Clinical assessments - Radiological examination with CBCT	Intentionally exposed ACM is as effective as dPTFE in ridge preservation. Furthermore, the use of ACM may help to reduce postoperative VAS scores and improve the quality of bone available for implant placement, as demonstrated by improved histomorphometric measures.	3 months

**Table 6:** Studies on the use of amniotic membranes in the treatment of medication-related osteonecrosis of the jaw.F—female, M—male, MRONJ—medication-related osteonecrosis of the jaw, CAM—cryopreserved amniotic membrane, CBCT—cone beam computer tomography, VAS—visual analog scale, BRONJ—bisphosphonate-related osteonecrosis of the jaw.

Author Year	Patients	Sex	Age Range	Indications	Treatment	Evaluation Methodology	Results	Follow Up Period
Çanakçiet al., 2022 [47]	5	2F 3M	42–82	MRONJ stage 2–3	CAM	- CBCT - Level of mucosal coverage	- There was no recurrence of the infection and pain during follow-up sessions. - Complete mucosal closure was achieved in 6 necrosis sites. In only 1 patient, mucosal coverage was not achieved.	3 months–3 years
Ragazzo et al., 2022 [48]	26/23	20F 6M	Mean 69.48	MRONJ stage 1–3	(1) CAM (2) exclusively with restrictive surgery	- Pain on the VAS - Clinical assessments - Radiological examination	- Pain reduction - Stimulation of soft tissue healing	24 months
Odet et al., 2022 [49]	8	6F 2M	49–88	MRONJ stage 2–3	CAM	- Pain on the VAS - Clinical assessments - Radiological examination	- Overall, 80% of lesions had complete or partial wound healing. - No symptoms of infections in all patients - Pain relief in all patients	6 months
Val et al., 2021 [50]	26	21F 6M	36–89	MRONJ	CAM	- Pain on the VAS - Clinical assessments - Radiological examination	- Thirty days after their surgical treatment, only 2 patients showed persistent bone exposure. Both patients were successfully retreated. - Within 7 days postoperation, 92.5% of patients no longer experienced pain.	24 months
Ragazzo et al., 2018 [51]	2	1F 1M	85	Bisphosphonate-related osteonecrosis of the jaw (BRONJ)	CAM	- Clinical assessments - Radiological examination	- Wound was completely sealed. - Patients were asymptomatic. - No further abscesses developed.	6 months

**Table 7:** Studies on the use of amniotic membranes in the repair of nasal septal perforations. F—female, M—male, NSP—nasal septal perforations, AM—amniotic membrane.

Author Year	Patients	Sex	Age Range	Indications	Treatment	Evaluation Methodology	Results	Follow Up Period
Farhadi Shabestariet al., 2022 [52]	12	4F 8M	26–40	NSPs	Mucosal rotational flap + Cryopreserved AM	Clinical assessments	Successful repair was observed in 10 out of 12 patients (83%). Two patients experienced reperforation, but the size of the defect was smaller than the original. All patients reported the elimination of NSP-associated symptoms.	3 months
